# Unrevealing the multitargeted potency of 3-1-BCMIYPPA against lung cancer structural maintenance and suppression proteins through pharmacokinetics, QM-DFT, and multiscale MD simulation studies

**DOI:** 10.1371/journal.pone.0303784

**Published:** 2024-06-21

**Authors:** Mohammed Ali Alshehri, Saeed A. Asiri, Nawal Helmi, Hanadi M. Baeissa, Abdullah Hamadi, Abdulrahman Alzahrani, Rashed Mohammed Alghamdi, Misbahuddin M. Rafeeq, Zeyad M. Alharbi, Mohammad Azhar Kamal

**Affiliations:** 1 Department of Clinical Laboratory Sciences, Faculty of Applied Medical Sciences, Najran University, Najran, Kingdom of Saudi Arabia; 2 Department of Biochemistry, College of Science, University of Jeddah, Jeddah, Kingdom of Saudi Arabia; 3 Department of Biological Science, College of Science, University of Jeddah, Jeddah, Kingdom of Saudi Arabia; 4 Department of Medical Laboratory Technology, Faculty of Applied Medical Sciences, University of Tabuk, Tabuk, Kingdom of Saudi Arabia; 5 Department of Applied Medical Sciences, Applied College, Al-Baha University, Al-Baha City, Kingdom of Saudi Arabia; 6 Department of Laboratory Medicine, Faculty of Applied College, Al-Baha University, Al-Baha City, Kingdom of Saudi Arabia; 7 Department of Pharmacology, Faculty of Medicine, Rabigh. King Abdulaziz University, Jeddah, Kingdom of Saudi Arabia; 8 Department of Pharmaceutics, College of Pharmacy, Prince Sattam Bin Abdulaziz University, Al-Kharj, Kingdom of Saudi Arabia; American University, ARUBA

## Abstract

Lung cancer, a relentless and challenging disease, demands unwavering attention in drug design research. Single-target drugs have yielded limited success, unable to effectively address this malignancy’s profound heterogeneity and often developed resistance. Consequently, the clarion call for lung cancer drug design echoes louder than ever, and multitargeted drug design emerges as an imperative approach in this landscape, which is done by concurrently targeting multiple proteins and pathways and offering a beacon of hope. This study is focused on the multitargeted drug designing approach by identifying drug candidates against human cyclin-dependent kinase-2, SRC-2 domains of C-ABL, epidermal growth factor and receptor extracellular domains, and insulin-like growth factor-1 receptor kinase. We performed the multitargeted molecular docking studies of Drug Bank compounds using HTVS, SP and XP algorithms and poses filter with MM\GBSA against all proteins and identified DB02504, namely [3-(1-Benzyl-3-Carbamoylmethyl-2-Methyl-1h-Indol-5-Yloxy)-Propyl-]-Phosphonic Acid (3-1-BCMIYPPA) as multitargeted lead with docking and MM\GBSA score range from -8.242 to -6.274 and -28.2 and -44.29 Kcal/mol, respectively. Further, the QikProp-based pharmacokinetic computations and QM-based DFT showed acceptance results against standard values, and interaction fingerprinting reveals that THR, MET, GLY, VAL, LEU, GLU and ASP were among the most interacting residues. The NPT ensemble-based 100ns MD simulation in a neutralised state with an SPC water model has also shown a stable performance and produced deviation and fluctuations <2Å with huge interactions, making it a promising multitargeted drug candidate—however, experimental studies are suggested.

## 1. Introduction

Lung cancer is a formidable adversary, demanding relentless attention and innovative solutions. It is a disease that has touched the lives of millions, causing immeasurable suffering and loss. Despite significant advances in medical research and treatment options, lung cancer is still one of the top causes of cancer-related fatalities worldwide [1, 2]. Its complexity and heterogeneity make it an exceptionally challenging target for drug development. In the quest to combat lung cancer, drug design research has evolved significantly over the years [1]. Early efforts primarily focused on developing single-target drugs. These drugs were designed to target specific proteins or pathways critical in the growth and progression of lung cancer cells. While they provided some benefits to specific patients, they often fell short of addressing the full spectrum of the disease. The limitations of single-target drugs became apparent as researchers grappled with lung cancer’s profound heterogeneity [2–5]. Lung cancer is not uniform; it encompasses various subtypes with unique genetic and molecular characteristics [6–8]. Moreover, lung cancer cells are notorious for their ability to adapt and develop resistance to targeted therapies. This adaptability has frustrated many drug development efforts, leaving patients with limited treatment options. In response to these challenges, a new approach, multitargeted drug design, has emerged, recognising the need to address the complexity of lung cancer by simultaneously targeting multiple proteins and pathways involved in its growth and progression. It seeks to broaden the scope of drug candidates, offering a more comprehensive and adaptable strategy. The multitargeted drug design approach has gained traction because it holds the potential to overcome some of the key limitations of single-target drugs [9–11]. Targeting multiple proteins or pathways can disrupt cancer cells’ ability to adapt and develop resistance and has the advantage of addressing the heterogeneity of lung cancer.

In this study, we focus on four proteins that play crucial roles in the context of lung cancer: human cyclin-dependent kinase-2 (CDK2), SRC-2 domains of C-ABL, epidermal growth factor (EGF), and insulin-like growth factor-1 receptor kinase (IGF-1R). These proteins are involved in essential signalling pathways that regulate cell growth, proliferation, and survival. Dysregulation of these pathways is often observed in lung cancer, making them attractive targets for drug development [12–15]. CDK2 is a crucial protein involved in cell cycle regulation, and the dysregulation of the cell cycle is common in lung cancer. CDK2 can become overactive, leading to uncontrolled cell division and tumour growth in lung cancer patients. Targeting CDK2 has been explored as a potential therapeutic strategy for lung cancer treatment [16]. The IGF-1R is a cell surface receptor involved in cell growth and proliferation, and overexpression is associated with lung cancer, as it can stimulate the growth and survival of cancer cells. Inhibiting IGF-1R signalling has been studied as a potential treatment approach for lung cancer patients [17]. The C-ABL gene can undergo genetic mutations that lead to the formation of an abnormal fusion protein called BCR-ABL, which is characteristic of a type of leukaemia (chronic myeloid leukaemia). While C-ABL mutations are not a primary driver of lung cancer, they are relevant in targeted therapy for specific lung cancer subtypes with overlapping genetic alterations [18]. The EGF and its receptor EGFR play a significant role in lung cancer. EGFR is often mutated or overexpressed in lung cancer, leading to uncontrolled cell growth and division. Targeted therapies such as EGFR inhibitors have been developed to target this pathway in lung cancer patients [19]. These proteins have distinct roles in lung cancer, but all are interconnected through various signalling pathways. The EGFR signalling can activate downstream kinases, including CDK2 and IGF-1R, leading to cell cycle progression and growth. Some lung cancer subtypes may exhibit multiple proteins or pathway alterations simultaneously, necessitating a multi-targeted therapeutic approach [20, 21]. Understanding the complex network of proteins and signalling pathways involved in lung cancer is crucial for developing effective treatments and personalized medicine approaches [22, 23]. We employ multiscale molecular dynamics simulations to explore the multitargeted drug design approach. This computational technique allows us to study the dynamic behaviour of molecules at various levels of detail, from individual atoms to larger molecular complexes. By simulating the interactions between our drug candidates and the target proteins, we can gain valuable insights into their binding affinities, mechanisms of action, and potential effectiveness [24]. In addition to MD simulations, we consider pharmacokinetics through the lens of ADMET (Absorption, Distribution, Metabolism, Excretion, and Toxicity). Understanding how drugs are absorbed, distributed, metabolised, and eliminated in the body is crucial for assessing their potential as therapeutic agents. We aim to identify drug candidates with favourable pharmacokinetic profiles by incorporating ADMET-based analyses [25–28]. Furthermore, we employ molecular interaction fingerprints to characterise and compare the interactions between our drug candidates and the target proteins. This approach provides a systematic way to assess our compounds’ binding patterns and specificity, aiding in selecting promising candidates [29–32].

Lung cancer is a relentless challenge that calls for innovative solutions, and multitargeted drug design offers a beacon of hope. Our study aims to contribute to this evolving field, ultimately bringing us closer to effective treatments where we aim to identify a multitargeted drug candidate that potentially can target all four proteins. We conducted the docking studies with three state-of-the-art algorithms- HTVS, SP and XP and docked poses were filtered with MM\GBSA and pharmacokinetics, and interaction fingerprints were performed to understand the pattern while the MD simulation to evaluate all the results and check whether the compounds are stable [33].

## 2. Methods

**Fig 1:**
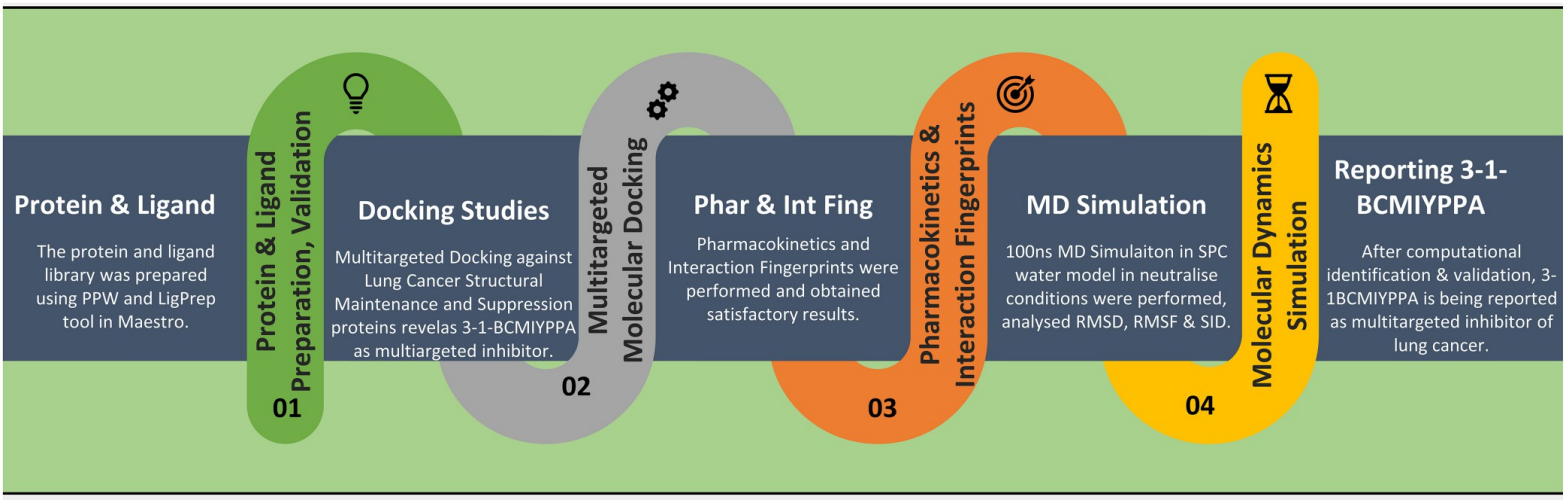
The graphical abstract of the study to identify lung cancer’s multitargeted inhibitor.

In Fig 1, we have shown the graphical abstract or the flow of the study to make it clear to understand, and the detailed methods are as follows-

### 2.1 Protein and ligand preparation and structural validation

Protein preparation is essential to ensure the accuracy and reliability of molecular simulations and drug binding studies. It involves filling in missing parts of the protein structure, optimising its conformation, and eliminating water molecules irrelevant to the binding interactions. Properly prepared proteins provide a realistic starting point for simulations, enabling researchers to study their behaviour accurately and assess drug-protein interactions effectively. This preparation ensures that the results obtained in computational studies are meaningful and biologically relevant [34]. We used the Protein Preparation Wizard (PPW) from Schrodinger Maestro (https://www.schrodinger.com/) to prepare the proteins and the structures were downloaded from https://www.rcsb.org/, the RCSB database [35–37]. The lung cancer structural maintenance and suppression proteins were found after searching the RCSB database. These proteins include cyclin-dependent kinase-2 (PDBID: 1AQ1), insulin-like growth factor-1 receptor kinase (PDBID: 1K3A), SRC-2 domains of C-ABL (PDBID: 1AB2), and epidermal growth factor and receptor extracellular domains (PDBID: 1IVO) [16–19]. We observed that PDBID: 1AQ1 and 1K3A both have ligands, Chain A of the proteins, and solvents, whereas PDBID: 1AB2 only has Chain A of the protein and PBDID: 1IVO has Chains A, B, C, and D of the proteins, as well as additional heteroatoms. The missing side chains and loops were filled in using Prime in the PPW’s (https://www.schrodinger.com/) preprocess tab, the termini were capped, oxygen was added to the termini, hydrogens were replaced, disulphide bonds and zero-bond orders to metals were created, possible states were generated using Epik at pH 7.4 2, and bond orders were assigned to the CCD database [33, 34, 38, 39]. As crystal structures were present in each condition, sample water orientation and crystal symmetry were employed in the optimisation tab to optimise the structures, and PROPKA (https://propka.readthedocs.io/en/latest/index.html) was used to optimise the protein structures at pH 7 [40]. We used an OPLS4 forcefield (https://www.schrodinger.com/), converged heavy atoms to RMSD 0.30, and deleted water beyond 5 to the ligand under the minimisation and delete waters tab [41]. In PDBID: 1AQ1 and 1K3A, we only kept ligands and Chain A after preparation, and in PDBID: 1AB2 and 1IVO, we only kept Chain A. In order to determine whether the protein’s residue was eventually in a satisfactory location, the structures were checked for errors and plotted on a Ramachandran plot. The LigPrep tool in Maestro (https://www.schrodinger.com/) was used to prepare the entire chemicals library from DrugBank (https://go.drugbank.com/) [36, 42–45]. The OPLS4 forcefield was used, and the maximum ligand size was limited to 500 atoms [41]. During ionisation, potential states at the desired pH of 7.2 were created using Epik (https://www.schrodinger.com/platform/products/epik/), along with metal-binding states, the original state, Desalt, and the creation of tautomers [38]. The output was written in SDF format, and the stereoisomer calculations generated 32 ligands from each compound while maintaining the desired chiralities.

### 2.2 Receptor Grid Generation (RGG) and molecular docking

Creating a receptor grid is crucial before docking studies to enhance the accuracy and efficiency of molecular docking simulations. The grid defines specific regions within the protein where potential ligands can bind, allowing precise predictions of binding affinities and orientations [46, 47]. By predefining the search space, docking algorithms can systematically explore ligand-protein interactions, significantly reducing computational time and resources. This preparation step ensures that docking studies focus on relevant binding sites, improving the reliability of drug discovery efforts and aiding in identifying potential drug candidates. The grids on each protein were created using Maestro’s Receptor Grid Generation (RGG) tool (https://www.schrodinger.com/), which is essential for molecular docking studies [36, 46, 48]. The grids on each protein were created using Maestro’s RGG tool, which is essential for molecular docking studies. In cyclin-dependent kinase-2 (PDBID: 1AQ1) and insulin-like growth factor-1 receptor kinase (PDBID: 1K3A), the ligand was already found and we selected the ligand site as active site and generated the grid whereas in the case of SRC-2 domains of C-ABL (PDBID: 1AB2), and Epidermal Growth Factor and Receptor extracellular domains (PDBID: 1IVO) we selected the complete proteins under the grid for blind docking studies. In the RGG tool, a scaling factor of 1.0 and partial charge cutoff of 0.25 was kept, whereas the site of the enclosing box was kept in the centroid of the workspace ligand where the native ligand was found, and the centroid of selected residue and all residues were selected where no native ligand was found, and size of the box was adjusted to adequately cover them while keeping all the advance options as default. For the docking-based screening of the compounds, we used the VSW tool that is Virtual Screening Workflow (https://www.schrodinger.com/), where the generated ligand library was retained as the ligand source and the library was merged to eliminate duplicates, distribute them for sub-jobs and identify unique compounds, resulting in a unique characteristic for each input molecule [46, 48]. We kept the QikProp (https://www.schrodinger.com/) filter that created the pharmacokinetic properties of the compounds in the filtering tab, prefiltered them using Lipinski’s rule based on the pharmacokinetic properties, and eliminated the ligands with reactive functional groups [36, 42, 49]. We skipped preparing the ligand because we had already prepared the library and browsed the grids file for the docking studies on the receptor tab. In the docking tab, the partial charge cutoff was preserved at 0.15, and the ligand van der Waals radii for nonpolar atoms were scaled with a scaling factor of 0.80. We also used the Epik state penalties for docking and wrote interaction scores for the residue within 12 of the grid centre [38]. The compounds were then screened using High Throughput Virtual Screening (HTVS), Standard Precision Docking and Extra Precise Docking (XP), followed by post-processing utilising Molecular Mechanics-based Generalized Born and Surface Area (MM\GBSA) [36, 46]. All 100% of the compound’s library was passed to HTVS, and then the top 10% of HTVS was passed to SP docking, and then the top 10% of SP was passed to XP docking, where we kept generating up to 4 poses per compound state and 100% of its data was passed to MM\GBSA to filter the poses [33, 36].

### 2.3 Pharmacokinetics and QM-based DFT studies

Pharmacokinetic properties refer to the processes by which the body absorbs, distributes, metabolises, and eliminates drugs. These properties determine how a drug behaves within an organism, including its bioavailability, half-life, and clearance. Understanding pharmacokinetics is vital because it helps optimise drug dosing regimens, predict drug interactions, and minimise adverse effects. By studying these properties, researchers and clinicians can ensure effective and safe drug therapies, tailoring treatments to individual patients and maximising the therapeutic benefits of pharmaceutical compounds. The pharmacokinetic properties of compound 3-1-BCMIYPPA were computed with the QikProp tool and compared with the standard values of the tool to check if the compound is appropriately fitting as a drug candidate [36, 42]. We utilized the Jaguar Optimization tool (https://www.schrodinger.com/) with specific settings to optimise our chosen ligand molecule [36, 50]. We employed the default B3LYP-D3 theory with a 6-13G*** basis set for Density Functional Theory, maintaining automatic SCF spin treatment. We set the SCF tab in the initial stage to ‘quick’ and ‘atomic overlap.’ For convergence criteria, we defined a maximum of 48 iterations, an energy change threshold of 5e-5 Hartree, and an RMS density matrix change threshold of 5e-06. We employed an SCF level shift of 0.0 Hartree to ensure stability and opted for no thermal smearing. The convergence scheme utilized was DIID, and we maintained consistency in orbital sets when dealing with isomers that shared the same basis set. We allowed 100 steps with default criteria in the optimisation tab and utilized Schlegel’s guess and redundant internal coordinates for the initial hessian. We selected all properties for property calculations, including vibration frequencies at thermochemistry conditions of 1.0 atm pressure and an initial temperature of 298.15K in Kal/mol units. Surface calculations involved electrostatic potential with no specified box size and an average local ionization energy of 5 pts/Å Kcal/mol. We also considered noncovalent interactions with a grid density of 20 pts/Å and focused on density and spin density. Molecular Orbitals were set from α HOMO-0 to LUMO+0 with two orbitals and β HOMO-0 to LUMO+0 with the same two orbitals. Solvation was addressed using the PBF model in a water solvent medium, and the output was saved in Gaussian format after the computations. We utilized the QM convergence monitor tool within the Jaguar module to evaluate the results, locating the job directory for analysis [36, 50].

### 2.4 Interaction Fingerprints

Molecular Interaction Fingerprints in protein-ligand complexes are structural patterns that encode information about noncovalent interactions, such as hydrogen bonding, van der Waals forces, and hydrophobic contacts. MIFs help identify and characterise key binding interactions between proteins and ligands. They are crucial for understanding the binding mode and strength, aiding in the rational design of drugs. MIF analysis provides insights into ligand selectivity, aiding in drug optimisation and lead compound identification, ultimately enhancing the efficiency of drug discovery processes. The Interaction Fingerprints tool in Maestro (https://www.schrodinger.com/) [36] was used to calculate the molecular interaction fingerprints, where we selected all protein-ligand complexes of cyclin-dependent kinase-2 (PDBID: 1AQ1) in complex with 3-1-BCMIYPPA, SRC-2 domains of C-ABL (PDBID: 1AB2) in complex with 3-1-BCMIYPPA, Epidermal Growth Factor and Receptor extracellular domains (PDBID: 1IVO) in complex with 3-1-BCMIYPPA, and insulin-like growth factor-1 receptor kinase (PDBID: 1K3A) in complex with 3-1-BCMIYPPA and aligned them all while keeping IK3A as reference and generated the fingerprints to display the matrix. In the interaction matrix, we only kept the interacting residues with any contact with ligand and coloured them to differential based on the position. We also kept the count of interacting residue and ligand interactions to identify the residues and check which complex interacted most.

### 2.5 Molecular Dynamics Simulation

Molecular Dynamics (MD) Simulations of protein-ligand complexes involve computer-based simulations that track the dynamic behaviour of molecules over time. This technique provides insights into how a ligand interacts with a protein, including conformational changes, binding stability, and energy profiles. MD simulations are essential for understanding these interactions’ complex, time-dependent nature, aiding drug design by predicting binding affinities, assessing drug stability, and informing structural modifications for enhanced drug-target interactions, ultimately expediting drug discovery and development. The Desmond package, free to download from https://www.deshawresearch.com, was used for the MD simulation investigations of the complexes developed by the D E Shaw Research team [36, 51]. The MD simulation retained the system setup and production run as separate processes. The system builder tool was used to create the systems file, keeping out ions and salt placement within 20Å, and neutralise complex by adding 4Cl^-^ in cyclin-dependent kinase-2 (PDBID: 1AQ1) in complex with 3-1-BCMIYPPA and Epidermal Growth Factor and Receptor extracellular domains (PDBID: 1IVO) and 2Cl^-^ in SRC-2 domains of C-ABL (PDBID: 1AB2) in complex with 3-1-BCMIYPPA and 18Na^+^ in insulin-like growth factor-1 receptor kinase (PDBID: 1K3A) in complex with 3-1-BCMIYPPA. In the solvation tab, a predefined SPC water model was used as a solute, and the orthorhombic box shape of the boundary was kept in 10 × 10 × 10 Å of shape and minimise the volume of the box to fix on the P-L complex properly, and we used the OPLS4 force field to run the jobs [41]. We ran the MD simulation production tasks using the Molecular Dynamics panel, setting the simulation time to 100ns and recording an interval of 100ps at an energy level of 1.2 to produce 1000 frames for each simulation job [36, 51]. Before the production run, we relaxed the system using the NPT-ensemble class at 300 K temperature and 1.01325 bar pressure [52]. The trajectories were analysed, and analytics data and charts were produced using the simulation interaction diagram (SID) tool.

## 3. Results

### 3.1 Protein structural analysis

**Fig 2:**
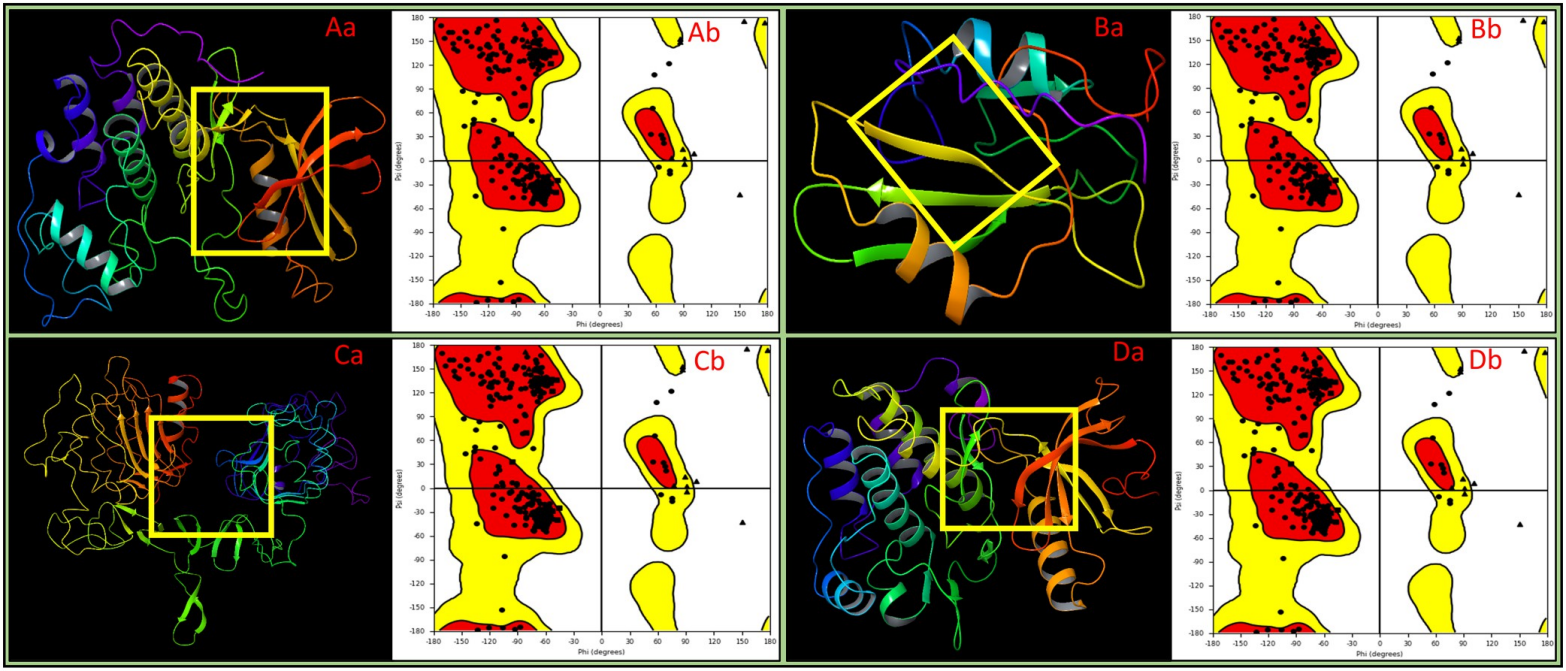
The prepared 3D structures of the proteins with ligand binding sites of **Aa)** 1AQ1, **Ba)** 1AB2, **Ca)** 1IVO, and **Da)** 1K3A. The Ramachandran plot for the prepared proteins shows that the structures are entirely acceptable for each case of **Ab)** 1AQ1, **Bb)** 1AB2, **Cb)** 1IVO, and **Db)** 1K3A.

The cyclin-dependent kinase-2 (CDK2), SRC-2 domains of C-ABL, Epidermal Growth Factor and Receptor extracellular domains, and insulin-like growth factor-1 receptor kinase (IGF-1R) were obtained from the RCSB database. We then used the Protein Preparation Wizard (PPW) from Schrodinger Maestro to prepare the proteins. The PPW is a comprehensive toolkit that allows us to perform various tasks, including adding hydrogens to the protein structure, assigning bond orders and atom types, removing water molecules and other irrelevant ligands, and optimising the protein structure for energy minimisation. After preparing the protein structures, we subjected them to rigorous validation that involved meticulously inspecting them for any anomalies or irregularities that could impact their stability or reliability. We employed the Ramachandran plot, a widely recognised tool for assessing the quality of protein structures, which is a two-dimensional graph that shows the distribution of phi (ϕ) and psi (ψ) dihedral angles for each amino acid residue in a protein. These angles define the rotation of the peptide bonds and are crucial for understanding the protein’s conformation. Ideally, most data points on the Ramachandran plot should fall within the favoured regions, indicating that the protein’s backbone conformation is energetically favourable and stable. Our analysis revealed that all protein residues fell within the Ramachandran plot’s satisfied region, affirming our prepared proteins’ structural integrity and stability. This rigorous preparation and validation process ensured that our proteins were in a biologically relevant state and exhibited structural stability, which is essential for the reliability of our subsequent molecular dynamics simulations and ligand binding studies. Our research aims to uncover the multitargeted potency of drug candidates against lung cancer-related proteins. The meticulous preparation and validation of protein structures lay the foundation for the subsequent stages of our research by ensuring that our proteins are in a state that is both biologically relevant and structurally stable. In all the protein cases, cyclin-dependent kinase-2 (PDBID: 1AQ1), SRC-2 domains of C-ABL (PDBID: 1AB2), Epidermal Growth Factor and Receptor extracellular domains (PDBID: 1IVO), insulin-like growth factor-1 receptor kinase (PDBID: 1K3A), the Ramachandran plot was flawlessly performed (Fig 2).

### 3.2 Protein-ligand interaction analysis

**Fig 3:**
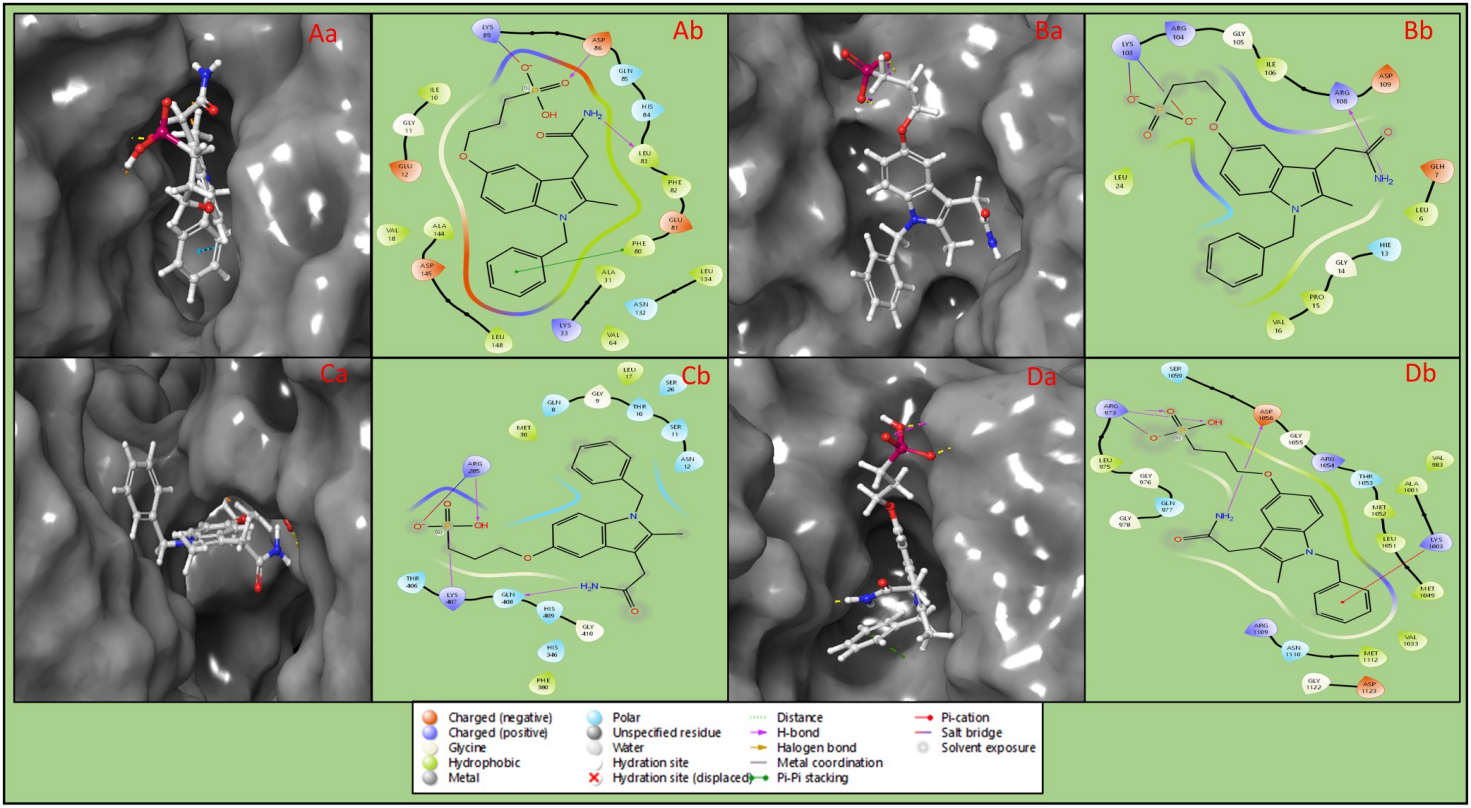
Showing the 3D docked best poses of **Aa)** 1AQ1, **Ba)** 1AB2, **Ca)** 1IVO, and **Da)** 1K3A, and 2D ligand interaction diagram of docked poses of **Ab)** 1AQ1, **Bb)** 1AB2, **Cb)** 1IVO, and **Db)** 1K3A and the legend is provided to understand bond and residue types.

Lung cancer signal transduction and cell growth proteins, namely cyclin-dependent kinase-2 (PDBID: 1AQ1), SRC-2 domains of C-ABL (PDBID: 1AB2), epidermal growth factor and receptor extracellular domains (PDBID: 1IVO), insulin-like growth factor-1 receptor kinase (PDBID: 1K3A) were docked with DrugBank compounds and identified 3-1-BCMIYPPA as multitargeted inhibitor for all the proteins. The interaction between the 3-1-BCMIYPPA and cyclin-dependent kinase-2 (PDBID: 1AQ1) has produced a docking score of -8.242 kcal/mol and MM\GBSA score of -43.56 kcal/mol by forming 2H-bonds contact among LEU83 with NH_2_ and ASP86 with O atom of the ligand. Additionally, it forms a salt bridge LYS89 with an O atom and a pi-pi stacking contact among PHE80 with the benzene ring of the ligand (Fig 3A and 3Ab, Table 1). The SRC-2 domains of C-ABL (PDBID: 1AB2) in complex with 3-1-BCMIYPPA have shown a docking score of -6.274 kcal/mol and MM/GBSA score of -28.20 kcal/mol by involving one hydrogen bond among ARG108 and NH_2_ atom and form 2 salt bridges among LYS103 and 2O atoms of the ligand (Fig 3B and 3Bb, Table 1). Interaction of human Epidermal Growth Factor and Receptor extracellular domains (PDBID: 1IVO) in complex with 3-1-BCMIYPPA showed docking score of -7.129 kcal/mol and MM/GBSA score of -44.29 kcal/mol by involving three hydrogen bonds among GLN408 with NH_2_ atom, ARG285 with OH atom, and LYS407 with O atom, and ARG285 forms a salt bridge with an O atom (Fig 3C and 3Cb, Table 1). The interaction between the insulin-like growth factor-1 receptor kinase (PDBID: 1K3A) in complex with 3-1-BCMIYPPA has produced the docking score of -6.847 kcal/mol and MM/GBSA score of -31.55 kcal/mol by involving three hydrogen bonds among ASP1056 and NH_2_ atom, ARG973 with O and OH atom. Additionally, ARG973 residue forms a salt bridge with an O atom and a pi-cation bond among LYS1003 with the benzene ring of the ligand (Fig 3D and 3Db, Table 1).

**Table 1:** Showing the docking score (Kcal/mol), MM\GBSA scores (Kcal/mol), and other important computations produced after multitargeted molecular docking studies.

SNo	PDBID	Docking Score	MMGBSA dG Bind	ligand efficiency sa	ligand efficiency ln	Evdw	Ecoul
1	1AQ1	-8.242	-43.56	-2.253	-9.975	-43.452	-10.225
2	1AB2	-6.274	-28.2	-1.458	-6.456	-28.428	-14.021
3	1IVO	-7.129	-44.29	-2.291	-10.142	-31.11	-13.512
4	1K3A	-6.847	-31.55	-1.632	-7.224	-34.258	-10.581

### 3.3 Pharmacokinetics and QM-based DFT studies

Pharmacokinetics is the study of how a drug is absorbed, distributed, metabolised, and excreted by the body over time. It involves understanding the drug’s fate within the body, its concentration levels in various tissues, and how the body processes it. In drug design, pharmacokinetics is crucial because it helps optimise a drug’s efficacy and safety. By comprehending how a drug behaves in the body, researchers can design molecules with desirable properties, such as prolonged duration of action, minimal side effects, and efficient delivery to target tissues. A deep understanding of pharmacokinetics ensures that drugs are effective in treating a specific condition, are well-tolerated, and have the potential for successful clinical outcomes, making them an essential component of drug development. Table 2 contains the descriptors, standard values, and pharmacokinetics properties of the compound 3-1-BCMIYPPA. The molecular weight of 3-1-BCMIYPPA (416.413 g/mol) falls within the acceptable range (130.0–725.0 g/mol), indicating that it is appropriately sized for drug design and the compound has 8.25 hydrogen bond acceptor groups, which is within the permissible range (2.0–20.0), suggesting that 3-1-BCMIYPPA may have suitable sites for interaction with other molecules. The computed lipophilicity values of 18.956 (QPlogPw) and 2.523 (QPlogPo/w) suggest that the compound exhibits moderate hydrophobicity, which falls within the acceptable range for drug-likeness (QPlogPw: 4.0–45.0; QPlogPo/w: 2.0–6.5). The compound has a PSA value of 128.095, within the acceptable range (7.0–200.0) and indicates a moderate polar surface area, potentially allowing for interactions with polar groups in biological targets. The compound is predicted to have a Human Oral Absorption value of 1, suggesting it has the potential for oral absorption. However, it is essential to note that the exact oral absorption behaviour may vary depending on other factors. The QPlogBB value of -2.697 indicates that the compound may not efficiently cross the blood-brain barrier, which could be advantageous if the intended therapeutic action does not require central nervous system penetration. With a value of -1.098, the compound is unlikely to pose concerns related to HERG channel binding, essential to avoid adverse cardiac effects. Both values (QPPCaco: 1.58, QPPMDCK: 1.55) fall within the acceptable ranges, suggesting favourable permeability characteristics, which are desirable for drug absorption. The compound contains 15 aromatic systems and 11 rotor bonds. These values are consistent with the range of 0–15 for aromatic systems and 0–15 for rotor bonds, indicating a moderately complex molecular structure. The compound complies with the Rule of Five (maximum of 4 violations) and the Rule of Three (maximum of 3 violations), indicating its potential as a drug candidate (Table 2). The computed properties of 3-1-BCMIYPPA suggest that it possesses several favourable characteristics for drug design, such as suitable size, hydrogen bond acceptors, and lipophilicity. Additionally, it demonstrates moderate polar surface area and permeability, which are vital for drug absorption. However, its limited BBB penetration and low likelihood of HERG channel binding make it a promising candidate for lung cancer.

**Table 2:** Showing the pharmacokinetics prediction of 3-1-BCMIYPPA and comparison with standard values of the QikProp tool.

Descriptor	Standard Values	3-1-BCMIYPPA	Descriptor	Standard Values	3-1-BCMIYPPA
Type	N/A	Small	HumanOralAbsorption	N/A	1
#acid	0–1	2	IP(eV)	7.9–10.5	8.031
#amide	0–1	1	Jm	N/A	0.014
#amidine	0	0	mol MW	130.0–725.0	416.413
#amine	0–1	0	%HumanOralAbsorption	>80% is high, <25% is poor	45.275
#in34	N/A	0	PISA	0.0–450.0	252.499
#in56	N/A	15	PSA	7.0–200.0	128.095
#metab	1–8	5	QPlogBB	−3.0–1.2	-2.697
#NandO	2–15	7	QPlogHERG	concern below −5	-1.098
#noncon	N/A	0	QPlogKhsa	−1.5–1.5	-0.703
#nonHatm	N/A	29	QPlogKp	−8.0 –−1.0	-4.103
#ringatoms	N/A	15	QPlogPC16	4.0–18.0	14.715
#rotor	0–15	11	QPlogPo/w	−2.0–6.5	2.523
#rtvFG	0–2	0	QPlogPoct	8.0–35.0	24.13
#stars	0–5	0	QPlogPw	4.0–45.0	18.956
accptHB	2.0–20.0	8.25	QPlogS	−6.5–0.5	-3.356
ACxDN^.5/SA	0.0–0.05	0.0228233	QPPCaco	<25 poor, >500 great	1.58
CIQPlogS	−6.5–0.5	-4.496	QPPMDCK	<25 poor, >500 great	1.55
CNS	−2 (inactive), +2 (active)	-2	QPpolrz	13.0–70.0	40.839
dip^2/V	0.0–0.13	0.0073203	RuleOfFive	maximum is 4	0
dipole	1.0–12.5	3.074	RuleOfThree	maximum is 3	1
donorHB	0.0–6.0	4	SAamideO	0.0–35.0	34.445
EA(eV)	−0.9–1.7	-0.03	SAfluorine	0.0–100.0	0
FISA	7.0–330.0	245.723	SASA	300.0–1000.0	722.945
FOSA	0.0–750.0	221.344	volume	500.0–2000.0	1290.483
glob	0.75–0.95	0.7929173	WPSA	0.0–175.0	3.379

**Fig 4:**
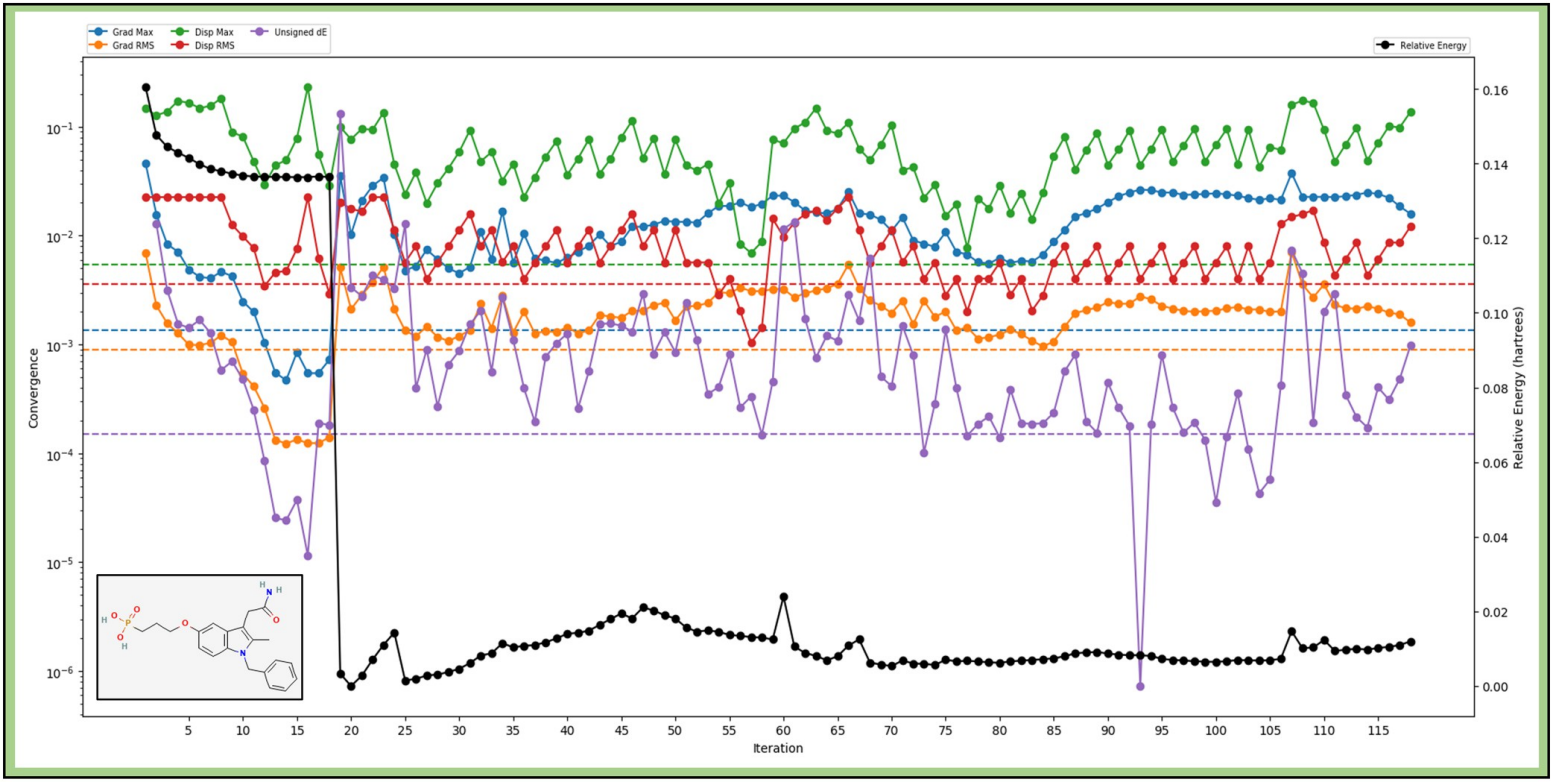
Showing the Quantum Mechanics-based Density Functional Theory optimisation of 3-1-BCMIYPPA where different energy levels are shown in different colours.

The computational analysis of 3-1BCMIYPPA using Density Functional Theory (DFT) with the B3LYP-D3 functional and a 6-31g** basis set has yielded detailed results. The molecule possesses 557 canonical orbitals in electronic structure, with the HOMO energy at -0.053769 Hartree and the LUMO at -0.006937 Hartree. The molecule exhibits a dipole moment of 37.3867 Debye, with components along the X, Y, and Z axes. Regarding vibrational properties, it displays a range of frequencies, from 25.961 cm^-1 to 3739.59 cm^-1, with no negative frequencies, indicating its stability (Fig 4). The zero-point energy is calculated to be 268.71 kcal/mol. At 298.15K and 1.00 atm, the molecule’s entropy is 171.653 Kcal/mol, enthalpy is 17.239162 kcal/mol, free energy is -33.93919 kcal/mol, and internal energy is 16.646677 kcal/mol. The heat capacity is 105.347 Kcal/mol/, and ln(Q) at these conditions is 57.283. In terms of total energy in atomic units, the internal energy is -1641.585286 au, the enthalpy is -1641.584342 au, and the free energy is -1641.6659 au. The electrostatic potential (ESP) analysis reveals the molecule’s charge distribution, with a minimum ESP of -144.99 kcal/mol, a maximum of 2.7 kcal/mol, and a mean ESP of -54.36 kcal/mol. The ESP local polarity is found to be 27.45 kcal/mol. Furthermore, the Atom-Least-Interaction Energies (ALIE) analysis provides insights into the noncovalent interactions within the molecule. The minimum ALIE is 67.81 kcal/mol, the maximum is 317.71 kcal/mol, and the mean ALIE is 210.95 kcal/mol. The average absolute deviation from the mean ALIE is 41.560254 kcal/mol. These comprehensive results offer valuable information for characterizing 3-1BCMIYPPA and its potential applications in drug design and related research endeavours. These extensive findings collectively contribute to a robust characterization of 3-1BCMIYPPA, opening avenues for potential lung cancer drug design applications.

### 3.4 Interaction Fingerprint analysis

**Fig 5:**
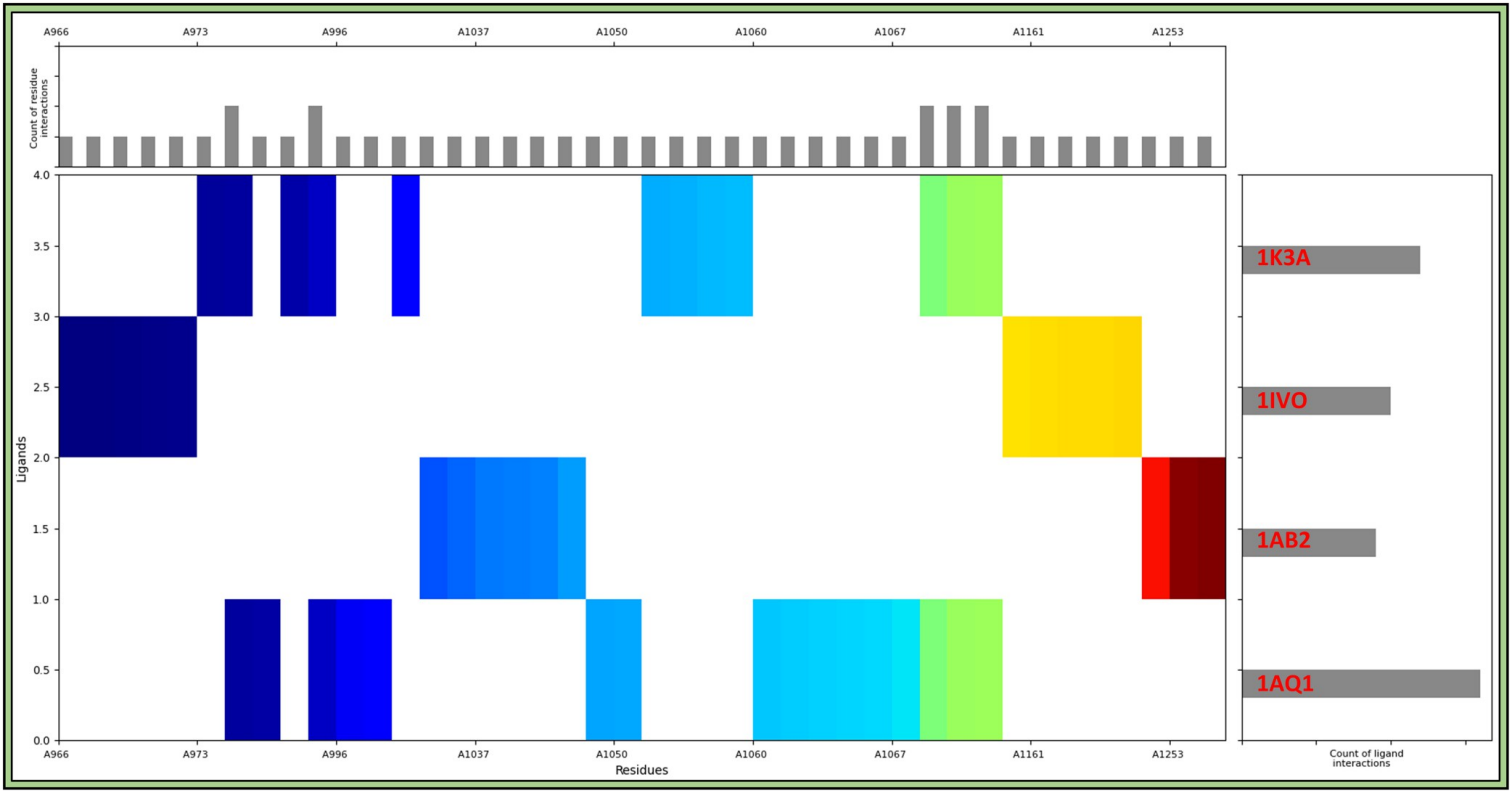
Molecular Interaction Fingerprints of 1AQ1, 1AB2, 1IVO, and 1K3A, where the main-coloured plot shows the N to C terminal of the protein, and the right-side plot shows the count of ligand interactions, and the upper plot shows the count of interacting residues.

Molecular Interaction Fingerprints in the context of protein-ligand complexes like Cyclin-Dependent Kinase-2, SRC-2 domains of C-ABL, human EGFR extracellular domains, and insulin-like growth factor-1 receptor kinase in complex with 3-1-BCMIYPPA are unique patterns that encode critical information about the interactions between the protein and ligand and represent the atomic contacts, hydrogen bonds, and noncovalent interactions. Analysing interaction fingerprints provides insights into how the ligand binds to the protein, helping understand the critical binding features and identifying essential interaction patterns contributing to binding affinity and specificity. The interaction matrix revealed a set of residues pivotal in binding 3-1-BCMIYPPA to these proteins. These count of residues and names are as follows- 5THR, 5MET, 5GLY, 4VAL, 4LEU, 4GLU, 4ASP, 3LYS, 3ARG, 2TYR, 2SER, 1PRO, 1ILE, 1HIS, and 1GLN (Fig 5). Residues such as 5THR, 5MET, 4VAL, and 4LEU are hydrophobic, which drive nonpolar molecules to aggregate in an aqueous environment, contribute significantly to ligand binding, and stabilise the complex. Residues like 4GLU, 4ASP, and 3LYS are polar and can involve hydrogen and electrostatic bonding. Residues such as 2TYR and 1HIS contain aromatic rings that can engage in π-π stacking interactions with aromatic or conjugated parts of ligands, contributing to the binding affinity and specificity and are known for their role in ligand recognition and stabilisation. Residues like 5GLY and 3ARG are relatively small and contribute to the flexibility and accommodation of ligands within the binding pocket, allowing for optimal ligand-protein interactions. Residues like 3ARG, 2SER, 1PRO, 1ILE, and 1GLN represent diverse residue types with unique properties. These residues may participate in various interactions, including hydrogen bonding, van der Waals forces, and more. The diverse set of interacting residues highlights the multi-faceted interactions between 3-1-BCMIYPPA and the target proteins, and the presence of hydrophobic, polar, and aromatic residues suggests a combination of a bond.

### 3.5 Molecular Dynamics Simulations

Molecular Dynamics Simulation is a computational technique that models the motion of atoms and molecules over time, and it simulates their interactions based on fundamental physics principles, allowing researchers to study the dynamic behaviour, conformational changes, and properties of molecular systems, providing valuable insights into their real-world behaviour. The system builder has generated 36106 atoms for 1AQ1, 19242 atoms for 1AB2, 70570 atoms for 1IVO, and 40198 atoms for 1K3A, which were taken for the 100ns production run. Further, we have analysed the trajectories of the simulation and extensively analysed RMSD, RMSF and intermolecular interactions formed during the simulations.

#### 3.5.1 Root Mean Square Deviation

**Fig 6:**
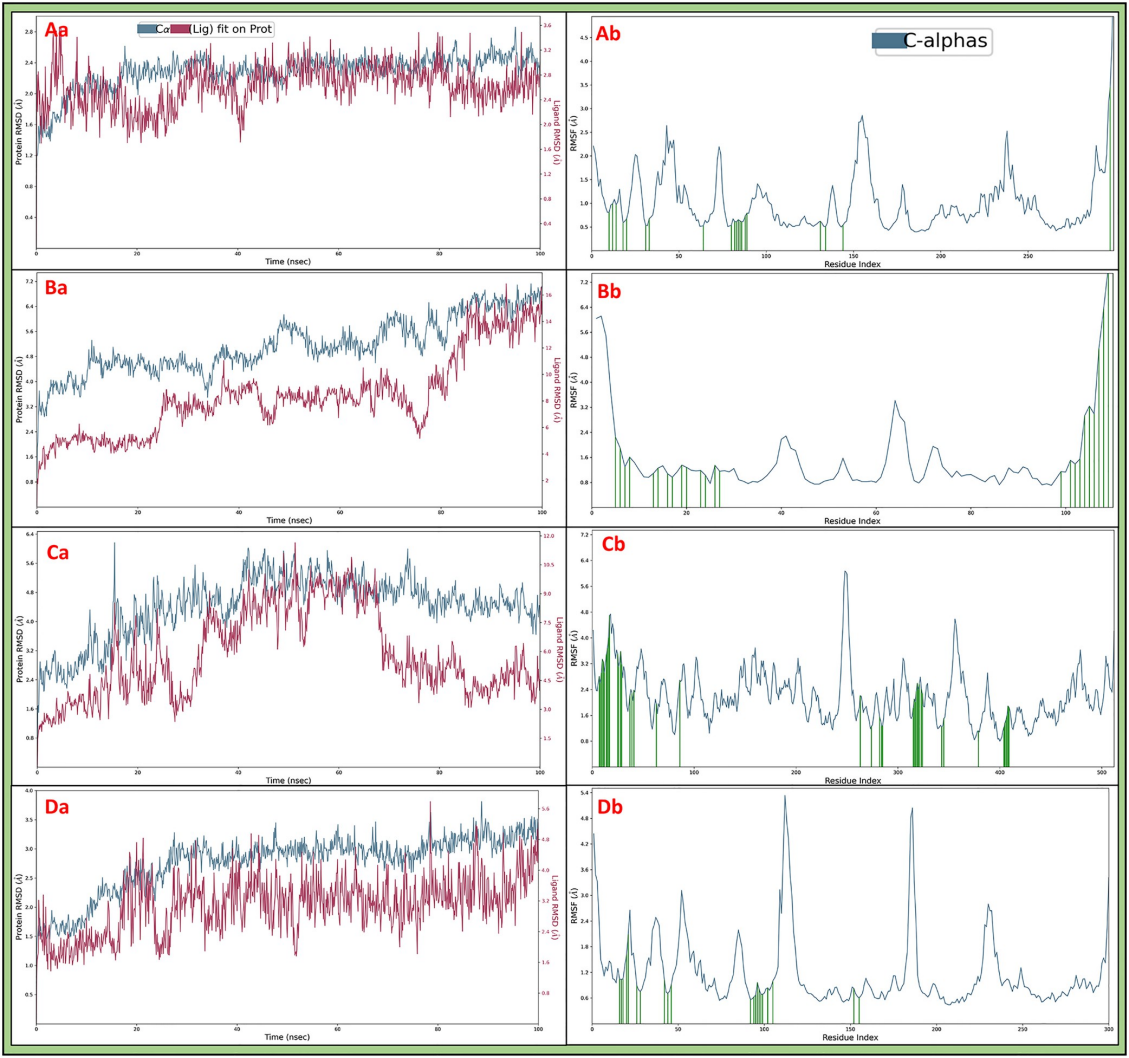
Showing the Root Mean Square Deviation (RMSD) of **Aa)** 1AQ1, **Ba)**1AB2, **Ca)** 1IVO, and **Da)**1K3A where red colour shows the deviation in 3-1-BCMIYPPA and blue shows in Cα, and Root Mean Square Fluctuations (RMSF) of **Ab)** 1AQ1, **Bb)**1AB2, **Cb)** 1IVO, and **Db)**1K3A, where blue colour shows the fluctuations in Cα, and green line shows 3-1-BCMIYPPA contacts.

Root Mean Square Deviation (RMSD) is a measure used in molecular modelling to assess the structural stability between protein and ligand, and it quantifies the average distance between corresponding atoms, indicating how much a model diverges from a reference structure. The cyclin-dependent kinase-2 (PDBID: 1AQ1) in complex with 3-1-BCMIYPPA initially deviated at 1.20 Å in the case of protein, while for the ligand case at 2.16 Å deviation was noted at 0.10 ns and after that point, the complete simulation shows a stable performance and at 100 ns, protein deviated to 2.29 Å while ligand deviated slightly to 2.83 Å. The deviation might result from adding ions to neutralise the system, sudden heat and ensemble class, and after neglecting the initial deviation, the RMSD of protein and ligand show acceptable performance (Fig 6Aa). The SRC-2 domains of the C-ABL (PDBID: 1AB2) in complex with 3-1-BCMIYPPA at the beginning deviated to 1.90 Å for protein, while the ligand deviated to 2.13Å at 0.10ns. However, at 100 ns, protein deviation was noted to be at 7.02 Å while ligand at 14.54 Å. This case was unique in that we found both protein and ligand kept deviating till 90ns and then tried to stabilise, and if initial deviation can be ignored or extended, it can show stability and trustability (Fig 6Ba). The human Epidermal Growth Factor and Receptor extracellular domains (PDBID: 1IVO) in complex with 3-1-BCMIYPPA produced protein deviation of 1.66 Å and ligand deviation of 1.45Å at 0.10 ns and at 100 ns, protein deviated till 4.44 Å, and ligand deviated till 3.98 Å. This deviation might result from sudden heat and change in the solute medium, and after ignoring the first 1ns, the whole RMSD displayed acceptable complex performance (Fig 6Ca). The insulin-like growth factor-1 receptor kinase (PDBID: 1K3A) in complex with 3-1-BCMIYPPA shows a deviation of 1.29 Å in the case of protein while 1.30 Å in the case of ligand at 0.10 ns and at 100 ns, the protein deviated to 3.17 Å and ligand to 4.20 Å and this case also shows a stable performance after ignoring the initial deviation that might be result of sudden heat, change in the solute medium (Fig 6Da). The RMSD in each condition has shown a stable performance, and it can be concluded that the complexes are stable after an initial few ns.

#### 3.5.2 Root Mean Square Fluctuations

Root Mean Square Fluctuation (RMSF) is a metric used in molecular dynamics simulations to analyse the flexibility of individual protein atoms. It calculates the average deviation of atomic positions from their mean positions over the simulation trajectory, revealing regions of high or low structural stability and flexibility. The cyclin-dependent kinase-2 (PDBID: 1AQ1) has shown a few fluctuating residues beyond 2Å are MET1, GLU2, LEU25, GLY43-THR47, GLU73, ASN74, GLY153-THR158, LYS237, PRO238, VAL289, and HIS295-LEU298 and many residues has formed interactions with 3-1-BCMIYPPA to make the complex stable are ILE10, GLU12, THR14, VAL18, LYS20, ALA31, LYS33, VAL64, PHE80, PHE82-LYS89, GLN131, LEU134, ALA144, and ARG297 (Fig 6Ab). The SRC-2 domains of C-ABL (PDBID: 1AB2) has shown a few fluctuating residues beyond 2Å are GLY1-SER5, SER40, SER41, ALA63, SER64-GLY66, and ARG104-ASP109 and many residues has formed interactions with 3-1-BCMIYPPA to make the complex stable are SER5-LYS8, HIS13, GLY14, VAL16, SER17, ASN19, ALA20, TYR23, LEU24, SER26, SER27, TYR99, and ALA101-ASP109 (Fig 6Bb). The Epidermal Growth Factor and Receptor extracellular domains (PDBID: 1IVO) has shown many fluctuating residues beyond 2Å are GLU2-CYS34, LEU38-THR57, GLY63, ALA68-ARG74, GLY85-TYR93, LEU98-LYS109, HIS121-ALA123, ASN128, PRO130, ALA131, ASN134, GLU136, SER137, GLN139, ARG141, ASP142, SER145, SER146-CYS207, THR217-ASP223, MET244-GLU258, GLY264-THR266, ASP279-GLY281, ALA289-SER291, GLU295-ASP297, LYS303-VAL312, GLY317-LEU325, ASN328-HIS334, LYS336, ASN337, PRO349, VAL350-ASP369, PRO387-THR391, GLY441, LYS443, SER460, GLY461, ILE467, SER468, ARG470-PRO488, PRO494-ARG497, and CYS502-VAL512, and many residues has formed interactions with 3-1-BCMIYPPA to make the complex stable are GLN8, GLY9, THR10, SER11, ASN12, LYS13, THR15, GLN16, LEU17, GLY18, SER26, LEU27, ARG29, MET30, LEU38, ASN40, GLU42, TYR64, MET87, GLY264, TYR275, CYS283, ARG285, ALA286, ILE316, GLY317, ILE318, GLY319, GLU320, PHE321, LYS322, SER324, LEU325, ASP344, HIS346, PHE380, ARG405, THR406, LYS407, GLN408, HIS409, GLY410 (Fig 6Cb). The insulin-like growth factor-1 receptor kinase (PDBID: 1K3A) has shown a few fluctuating residues beyond 2Å are VAL958-GLU961, GLY978, SER979, VAL992-PRO996, ALA1008-ARG1012, GLY1042, PRO1066-PRO1145, GLY1185-GLN1190, and LYS1256 and many residues has formed interactions with 3-1-BCMIYPPA to make the complex stable are ARG973, GLU974, LEU975, GLN977, GLY978, VAL983, GLU985, ARG999, ALA1001, LYS1003, MET1049, LEU1051, MET1052, THR1053, ARG1054, GLY1055, ASP1056, SER1059, ARG1062, ARG1109 and MET1112 (Fig 6Db).

#### 3.5.3 Simulation Interaction Diagrams

**Fig 7:**
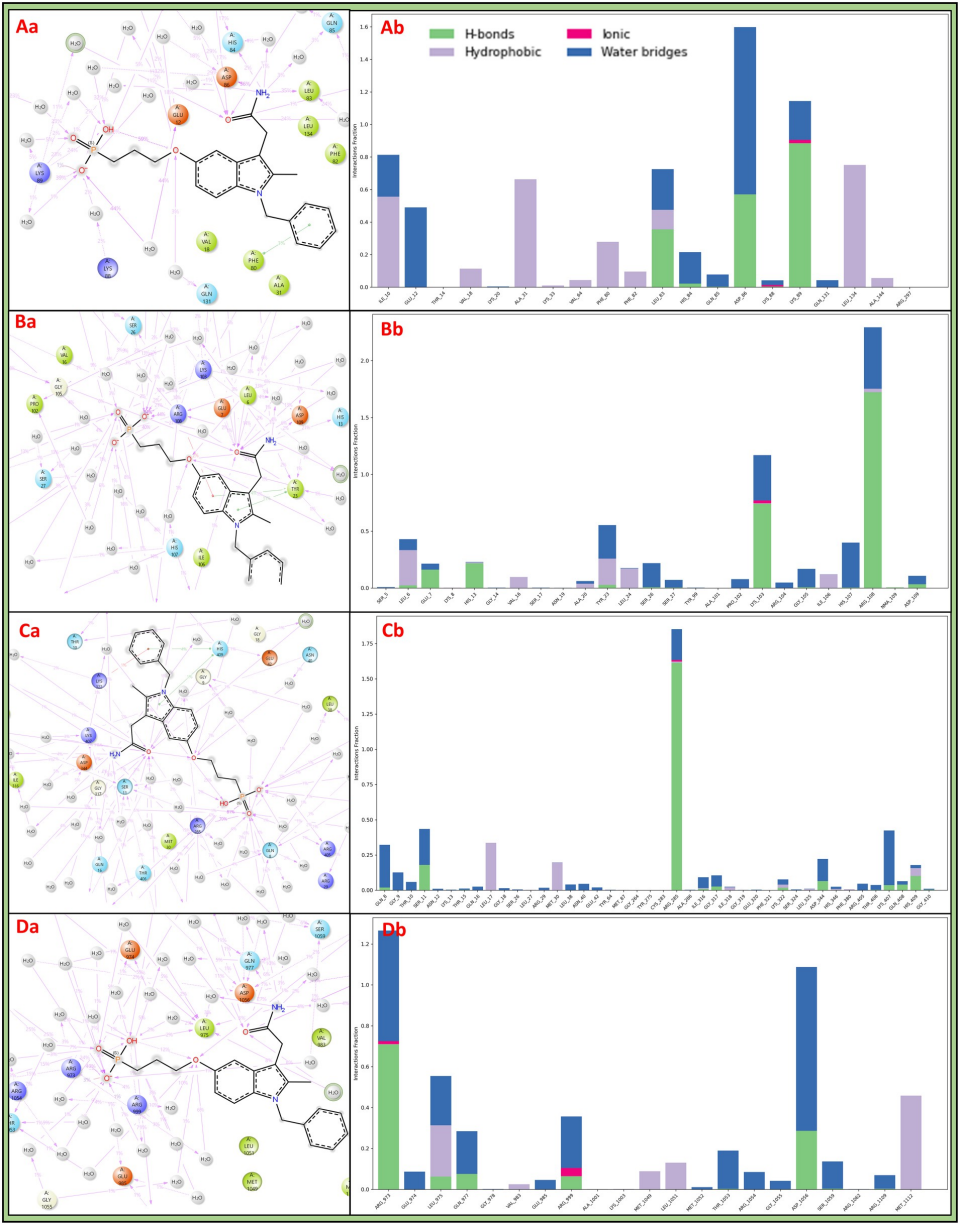
Showing the Simulation Interaction Diagram (SID) of **Aa)** 1AQ1, **Ba)**1AB2, **Ca)** 1IVO, and **Da)**1K3A and histogram representations of the count of interactions of **Ab)** 1AQ1, **Bb)**1AB2, **Cb)** 1IVO, and **Db)**1K3A, where blue colour shows water bridge, green shows H-bonds, red shows ionic interactions and grey shows hydrophobic interactions.

The Simulation Interaction Diagram (SID) is a visual representation used in molecular simulations to depict the dynamic interactions between proteins and ligands, and it illustrates how these molecules interact over time, showing the formation and breaking of bonds, hydrogen bonds, and noncovalent interactions, aiding in the analysis of molecular behaviours and properties. The cyclin-dependent kinase-2 (PDBID: 1AQ1) in complex with 3-1-BCMIYPPA involves thirteen water molecules that act as water bridges to provide stability and hydrogen bonds with LYS89 residue, and GLU12, ASP86, HIS84, LEU83, ILE10, and GLN131 residues with water molecules with 4O atoms, where NH_2_ atom with LEU83, ASP86 residues and GLN85 residue with water molecule, and OH atom interact with LYS89 residue (Fig 7Aa). The SRC-2 domains of C-ABL (PDBID: 1AB2) in complex with 3-1-BCMIYPPA interact many hydrogen bonds with HIS13, GLU7 residues and LYS103 residue with water molecule along NH_2_ atom, and four atoms interact along GLY105, LYS103, TYR23, PRO102, HIS107, ARG108 and SER26 residues with water molecules—additionally, a pi-pi stacking contact TYR23 with benzene ring of ligand (Fig 7Ba). The Epidermal Growth Factor and Receptor extracellular domains (PDBID: 1IVO) in complex with 3-1-BCMIYPPA formed more than fifteen water molecules act as water bridges and interaction with hydrogen bonds among GLN408, ASP344, SER11 residues and IlE316 residue with water molecule along with NH_2_ atom, ARG285 residue along OH atom, and 4O atoms contact HIS409, ARG285 residues and THR10, ASN40, MET30, GLN8, LYS407, SER11 and GLY317 residues with water molecules—also, one pi-pi stacking bond along HIS409 residue with the benzene ring of the 3-1-BCMIYPPA ligand (Fig 7Ca). The insulin-like growth factor-1 receptor kinase (PDBID: 1K3A) in complex with 3-1-BCMIYPPA involves more than 15 water molecules with water bridges. Hydrogen bonds interact among ASP1056, GLN977, and ARG1109 with water molecules with NH_2_ atom, while ARG999 and GLU974 with OH atom, and ARG973, ARG999 residues and ASP1056, GLN977, ARG1054, THR1053, SER1059 residues with water molecules (Fig 7Da). The simulation interaction diagram shows many interactions formed between the ligand 3-1-BCMIYPPA and proteins that are making the complexes stable, and it can be concluded that the computational studies are in complete support of treating 3-1-BCMIYPPA as a multitargeted drug candidate against lung cancer structural maintenance and suppression proteins. Further, we have shown the count of SID interactions in Fig 7Ab, 7Bb, 7Cb and 7Db to clarify which bonds form how many interactions for comparative visualisation.

## 4. Discussion

In our comprehensive study, we conducted molecular modelling analyses to identify potential drug candidates for targeting four key proteins involved in lung cancer: CDK2, SRC-2 domains of C-ABL, EGFR, and IGF-1R kinase. We aimed to discover multitargeted inhibitors and assess their pharmacokinetic properties and protein-ligand interactions. Molecular dynamics simulations were also performed to understand the dynamic behaviour of protein-ligand complexes. These results offer valuable insights for drug development. The initial step involved preparing and validating protein structures for accuracy. We used the PPW and structures from the RCSB database. Tasks included filling missing side chains, capping termini, adding hydrogens, creating disulfide bonds, and assigning orders. The Ramachandran plot analysis confirmed the stability and biological relevance of the prepared protein structures, ensuring their suitability for subsequent studies. We conducted molecular docking studies following protein preparation to identify potential drug candidates. 3-1-BCMIYPPA emerged as a promising multitargeted inhibitor for all four proteins. CDK2 displayed a strong binding affinity with multiple hydrogen bonds, salt bridges, and pi-pi stacking interactions, suggesting its potential as a drug candidate against CDK2 in lung cancer. SRC-2 domains of C-ABL also exhibited favourable binding, with hydrogen bonding and salt bridges. EGFR showed strong binding, forming multiple hydrogen bonds and a salt bridge with key residue, and IGF-1R demonstrated a potent interaction with 3-1-BCMIYPPA, including hydrogen and pi-cation bonds.

The properties of 3-1BCMIYPPA, obtained through rigorous computational analysis, offer valuable insights for drug design. The computational structure parameters, including HOMO and LUMO energy levels, provide crucial information about the molecule’s reactivity and potential interactions with target biomolecules. This knowledge serves as a foundation for designing compounds with specific pharmacological activities. Additionally, the dipole moment and polarizability of 3-1BCMIYPPA influence its solubility, membrane permeability, and pharmacokinetics, making them essential considerations in designing drug candidates. Furthermore, the vibrational frequencies and thermodynamic data aid in understanding the molecule’s stability and behaviour under different conditions, which are pivotal in optimizing drug formulations. These properties collectively guide the rational design of pharmaceutical agents with enhanced efficacy and minimized side effects, advancing the field of drug discovery and development. Understanding the pharmacokinetic properties of drug candidates is crucial, and computational analysis indicated favourable pharmacokinetics for 3-1-BCMIYPPA, supporting its potential as a lung cancer drug. Moreover, it displayed low toxicity potential for skin irritation and sensitisation, which is important for formulations. We performed interaction fingerprint analysis to delve deeper into interactions, highlighting specific interaction types contributing to ligand binding. Molecular dynamics simulations revealed the stability of protein-ligand complexes over time. The CDK2-3-1-BCMIYPPA complex remained stable, as did the SRC-2 domains of C-ABL. EGFR and IGF-1R complexes exhibited similar stability. Our simulation results showed that the CDK2-3-1-BCMIYPPA complex remained stable throughout the trajectory, with minimal deviations in the RMSD values, suggesting 3-1-BCMIYPPA forms a robust and stable complex with CDK2. In the case of SRC-2 domains of C-ABL, the RMSD values also indicated stability, with the complex maintaining its overall structure. EGFR and IGF-1R complexes exhibited similar stability during the simulations, with only minor fluctuations in RMSD values. These results demonstrate that 3-1-BCMIYPPA can form stable complexes with both EGFR and IGF-1R, which is crucial for its efficacy as a drug candidate.

RMSD measures the stability and structural changes of proteins and ligands during simulations. This study considered different protein-ligand complexes: 1AQ1, 1AB2, 1IVO, and 1K3A. The complex involving cyclin-dependent kinase-2 (CDK-2) and 3-1-BCMIYPPA showed initial deviations, likely due to added ions for system neutralisation and sudden heat. However, the protein and ligand demonstrated stable performance after this initial phase. In the case of the SRC-2 domains of C-ABL in complex with 3-1-BCMIYPPA, both the protein and ligand showed deviations until 90 ns and then attempted to stabilize. This unique behaviour suggests that stability and trustworthiness can be achieved if the initial deviation is neglected. The complex involving the extracellular domains of the human Epidermal Growth Factor and Receptor (EGFR) with 3-1-BCMIYPPA displayed initial deviations attributed to sudden heat and changes in the solute medium. However, after the initial 1ns, the complex exhibited acceptable performance. The insulin-like growth factor-1 receptor kinase (IGF-1R) in complex with 3-1-BCMIYPPA showed initial deviations, likely due to sudden heat and changes in the solute medium. After the initial phase, the complex displayed stable performance. In all cases, the RMSD analysis indicated that the complexes were stable after the initial few nanoseconds, suggesting that 3-1-BCMIYPPA has the potential to interact effectively with these proteins. RMSF analyses the flexibility of individual amino acid residues in proteins during simulations. This analysis provided insights into which residues fluctuated significantly and which formed stable interactions with 3-1-BCMIYPPA. CDK-2 in complex with 3-1-BCMIYPPA showed fluctuating residues, such as MET1, GLU2, LEU25, GLY43-THR47, GLU73, ASN74, GLY153-THR158, LYS237, PRO238, VAL289, and HIS295-LEU298. However, many residues formed stable interactions with the ligand, contributing to complex stability. The SRC-2 domains of C-ABL exhibited fluctuating residues like GLY1-SER5, SER40, SER41, ALA63, SER64-GLY66, and ARG104-ASP109. However, several residues formed stable interactions with 3-1-BCMIYPPA, contributing to complex stability. The EGFR extracellular domains showed fluctuating residues, but many residues formed stable interactions with the ligand. IGF-1R displayed fluctuating residues, but like the other complexes, it had stable interactions with 3-1-BCMIYPPA. The RMSF analysis highlighted that many residues interacted with 3-1-BCMIYPPA despite fluctuations to maintain complex stability. Interaction diagrams were used to understand how 3-1-BCMIYPPA interacts with water molecules and amino acid residues in the protein complexes. The CDK-2 complex involved water molecules forming bridges and hydrogen bonds with specific residues, providing stability. The C-ABL complex formed hydrogen bonds and pi-pi stacking interactions between the ligand and key residues. The EGFR complex had numerous water bridges and hydrogen bonds with various residues, ensuring complex stability. The IGF-1R complex displayed similar interactions with water molecules and residues. In all cases, these interaction diagrams highlighted the strong interactions between 3-1-BCMIYPPA and the proteins, supporting the compound’s potential as a multitargeted drug candidate against lung cancer structural maintenance and suppression proteins. The comprehensive analysis of molecular dynamics simulations, RMSD, RMSF, and interaction diagrams suggests that 3-1-BCMIYPPA can potentially effectively target and interact with various lung cancer-related proteins. Our study lays a strong foundation for experimental validation and drug development. The compound 3-1-BCMIYPPA’s promising binding affinities, favourable pharmacokinetics, and stability in protein-ligand interactions position it as a compelling candidate for further exploration, and our study brings us closer to developing effective therapies for lung cancer, addressing a global concern.

## 5. Conclusion

Our comprehensive study identified 3-1-BCMIYPPA as a highly promising multitargeted inhibitor with significant potential for treating lung cancer. The multitargeted molecular docking results consistently demonstrated strong binding affinities between 3-1-BCMIYPPA and crucial target proteins implicated in lung cancer progression, including CDK2, SRC-2 domains of C-ABL, EGFR, and IGF-1R and the robust interactions suggest the compound can effectively modulate the activity of these proteins, play pivotal roles in lung cancer development and growth. The predictions of pharmacokinetic low toxicity, including hepatotoxicity, cardiotoxicity, mutagenicity, skin irritation, and skin sensitisation, are highly encouraging. Moreover, interaction fingerprints shed light on the importance of hydrogen bonds, hydrophobic interactions, and pi-cation bonds in stabilising the complexes. The simulations further confirmed the stability and structural integrity of the complexes over time, supporting the potential of 3-1-BCMIYPPA as a multitargeted lung cancer inhibitor—however, experimental validation is suggested.

## Supporting information

S1 File (XLSX)

## Abbreviations

3-1-BCMIYPPA: [3-(1-Benzyl-3-Carbamoylmethyl-2-Methyl-1h-Indol-5-Yloxy)-Propyl-]-Phosphonic Acid
ADMET: Absorption, Distribution, Metabolism, Excretion, and Toxicity
ALIE: Atom-Least-Interaction Energies
CDK2: cyclin-dependent kinase-2
DFT: Density Functional Theory
EGF: epidermal growth factor
ESP: electrostatic potential
HTVS: High Throughput Virtual Screening
IGF-1R: insulin-like growth factor-1 receptor kinase
MD: Molecular Dynamics
MM\GBSA: Molecular Mechanics-based Generalized Born and Surface Area
PPW: Protein Preparation Wizard
RGG: Receptor Grid Generation
RMSD: Root Mean Square Deviation
RMSF: Root Mean Square Fluctuation
SID: Simulation Interaction Diagram
SP: Standard Precision
XP: Extra Precise Docking
ϕ: phi
ψ: psi
